# Supplementary material for: Thirteen Independent Genetic Loci Associated with Preserved Processing Speed in a Study of Cognitive Resilience in 330,097 Individuals in the UK Biobank
Source: Genes (Basel). 2022 Jan 10;13(1):122. doi: 10.3390/genes13010122 (PMC8774848; doi:10.3390/genes13010122)

Table of contents

		Page
Supplementary Figure S1	GWAS outputs	2
Supplementary Figure S2	Heat map of genetic correlations	4
Supplementary Figure S3	Plot of 13 genetic loci	5
Supplementary Figure S4	Circos plots of associated chromosomes	12
Supplementary Figure S5	Gene mapping of RT and comparison to *Resilience*	17

Supplementary Figure S1: Manhattan plots for (a) EY+Res, (b) EY/NonRes, (c) EY, (d) RT (n=164,000) (e) RT (n =333,664) (f) EduYears (g) Manhattan plot and QQ plot for cognitive change in the Health and Retirement Study. EY+Res and EY/NonRes were the input GWASs for GWAS-By-Subtraction (GBS) that generated the *Resilience* GWAS and the *EduYears* GWAS.

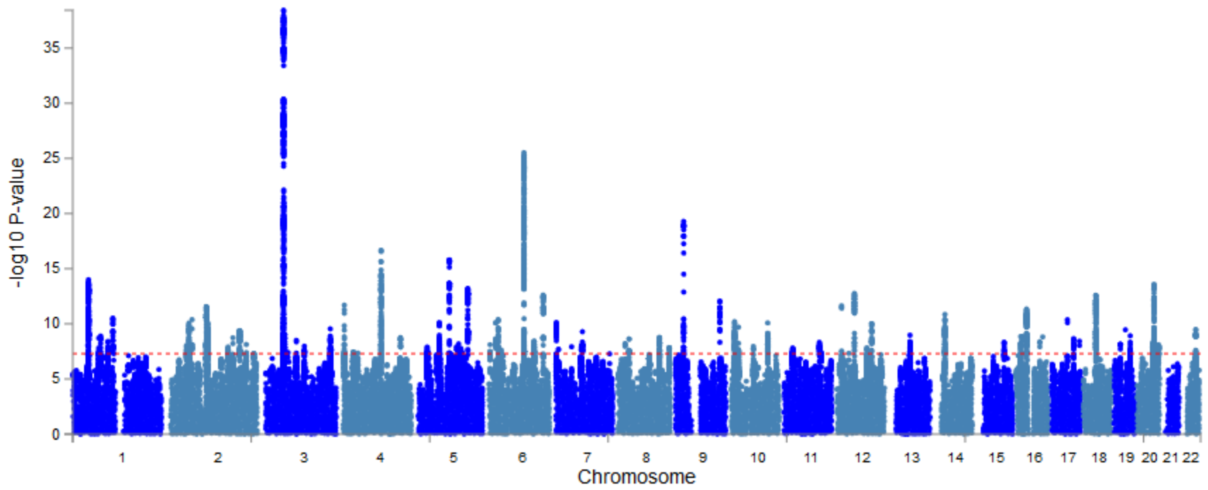

a. EY+Res (n = 156,011)

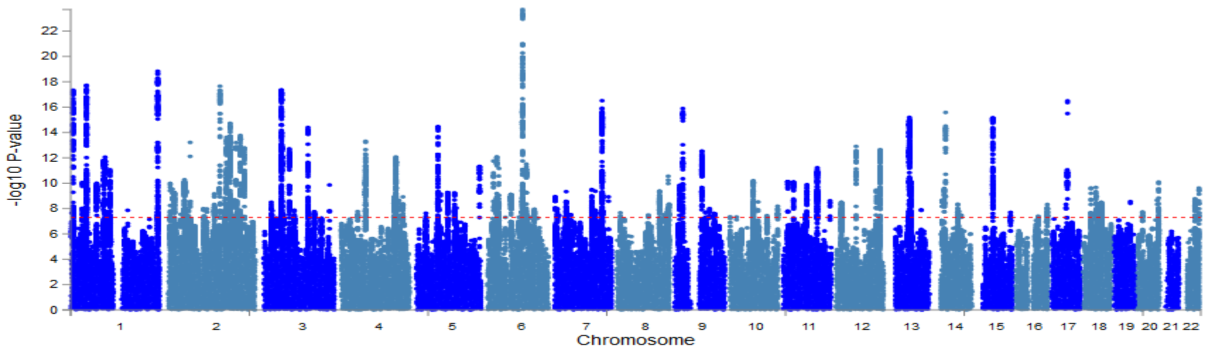

b. EY/NonRes (n = 174,086)

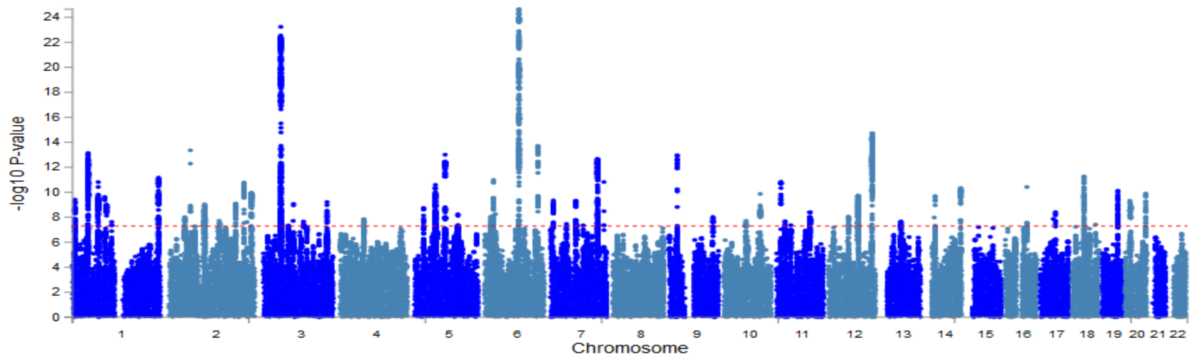

c. EY (n = 164,000)

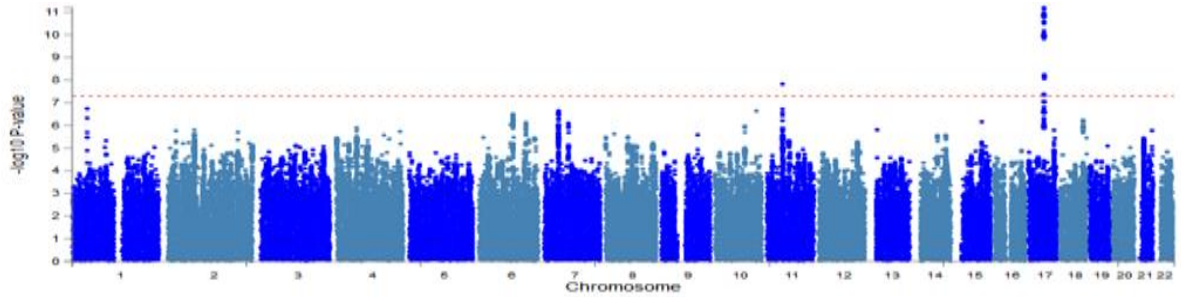

d. RT (n = 164,000)

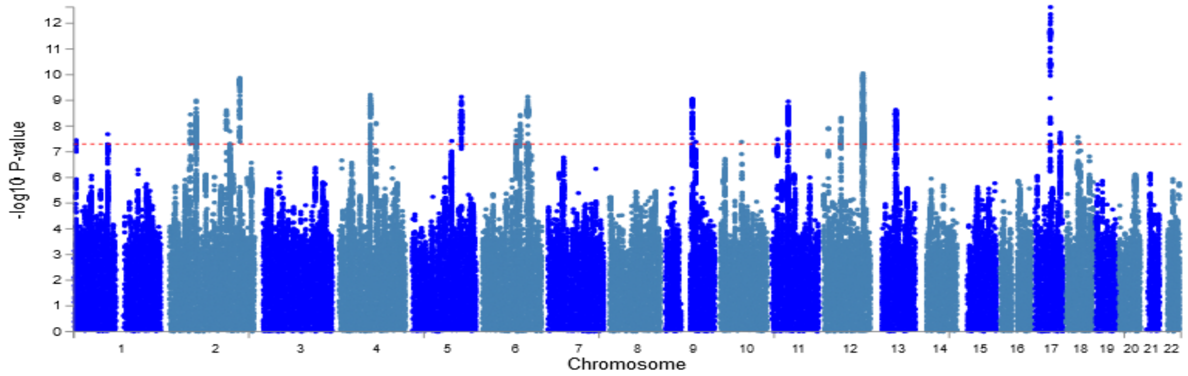

e. RT (n = 333,664)

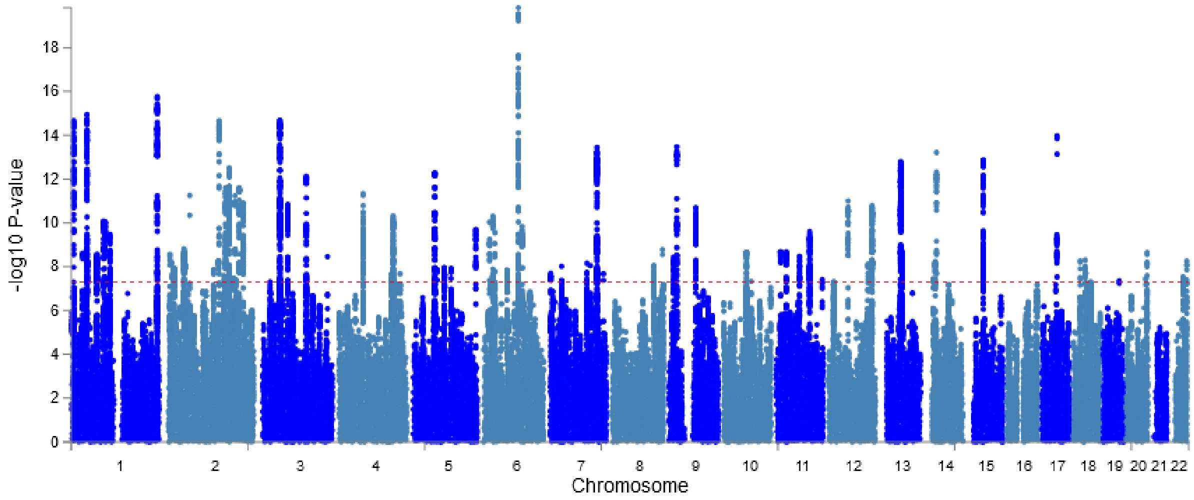

f. *EduYears* (n = 166,122)

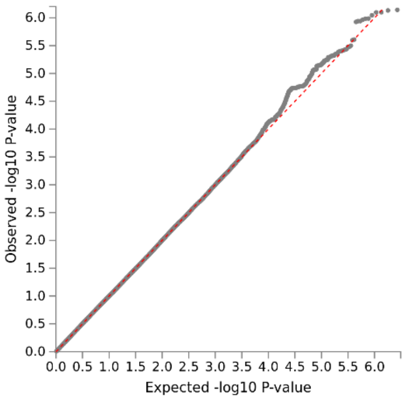

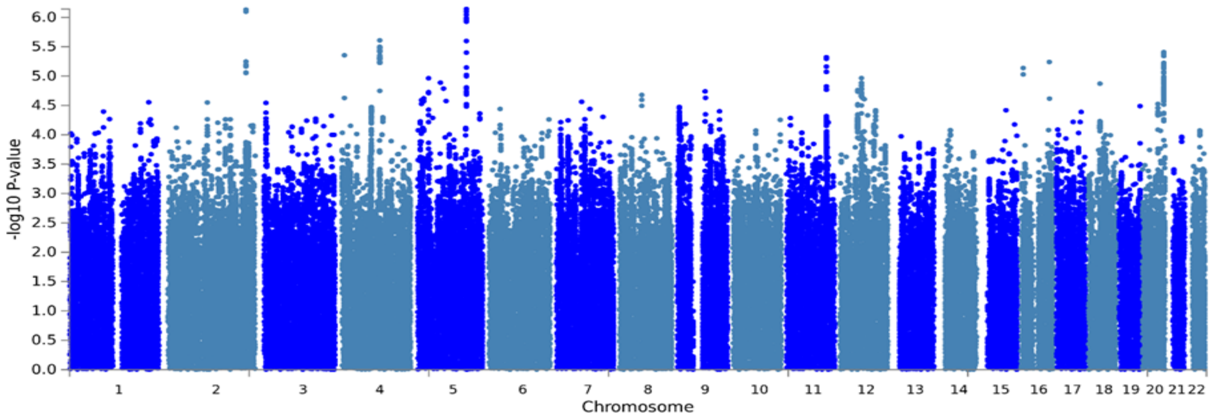

g. QQ plot and Manhattan plot of cognitive change in the Health and Retirement Study.

Supplementary Figure S2: *Heat map showing genetic correlations between the two GBS GWAS of Resilience and EduYears, the two inputs to GBS (EY+Res and EY/NonRes) and GWAS of the two variables used to create these phenotypes (EY and RT).*

	Resilience	*EduYears*	EY+Res	EY/NonRes	EY	RT
Resilience						
EduYears	0.01*					
EY+Res	0.84 ***	0.55***				
EY/NonRes	0.01*	1.0***	0.54***			
EY	-0.47***	-0.9***	-0.88***	-0.89***		
RT	0.8***	-0.56***	0.36***	-0.56***	0.14**	

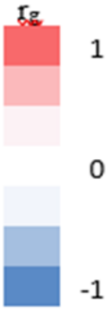

*** $P < 1 \times 10^{-10}$, ** $P < 0.0002$, * Not significant.

Supplementary Figure S3: Plots of the 13 independent genetic loci (a - m) showing the top lead SNPs, lead SNPs, independent significant SNPs and associated genes.

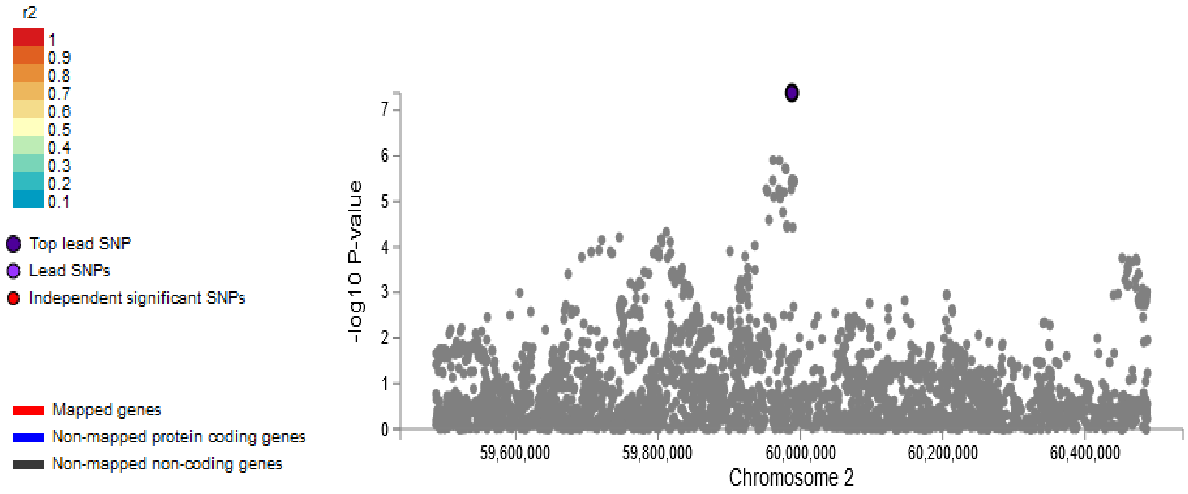

Locus 2: Chr2:59987310-59988258

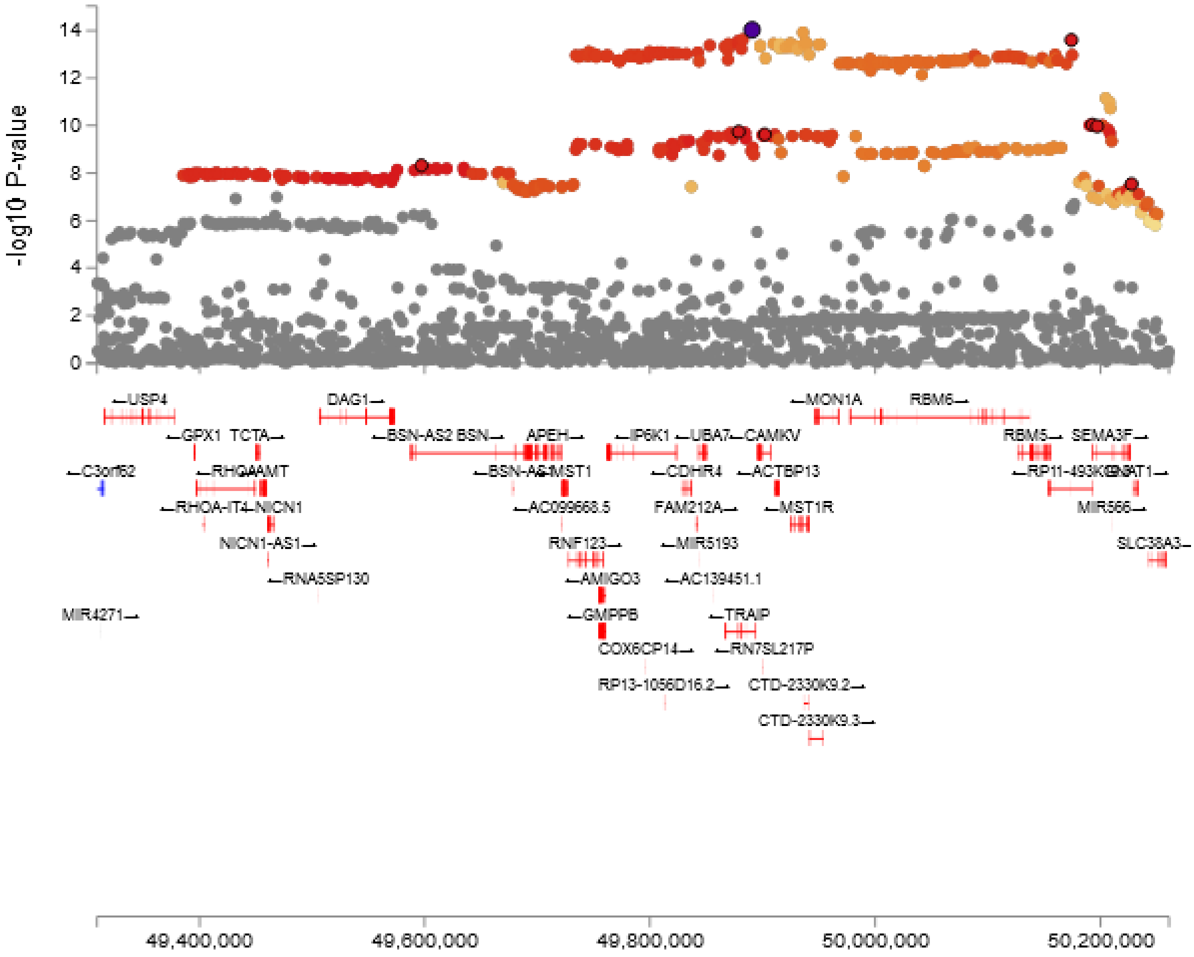

a. Locus 3: Chr3:49385417-50248954

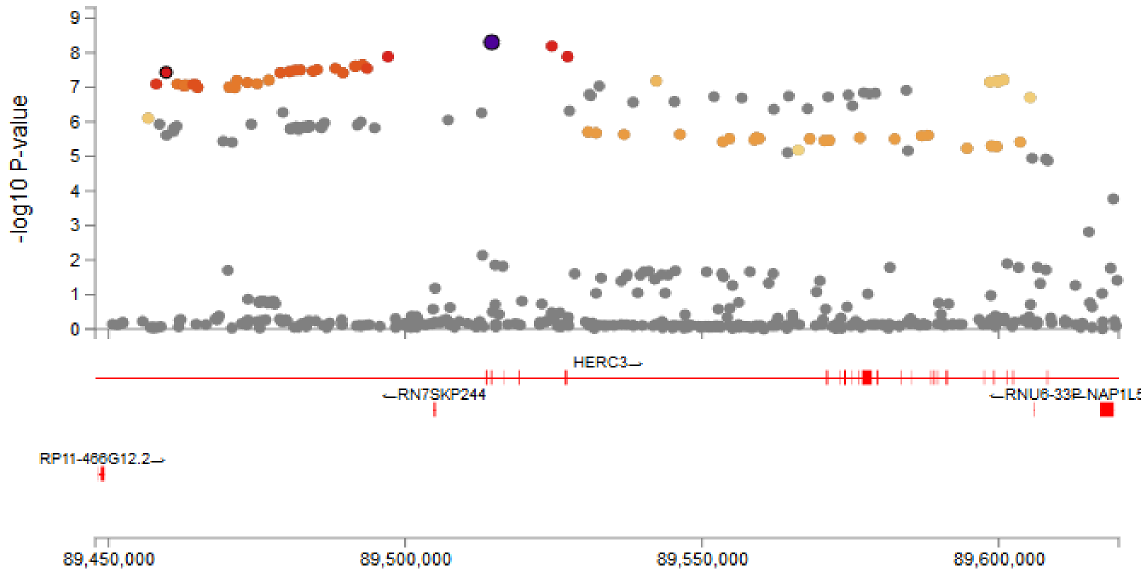

b. Locus 4A: Chr4:89455635-89612380

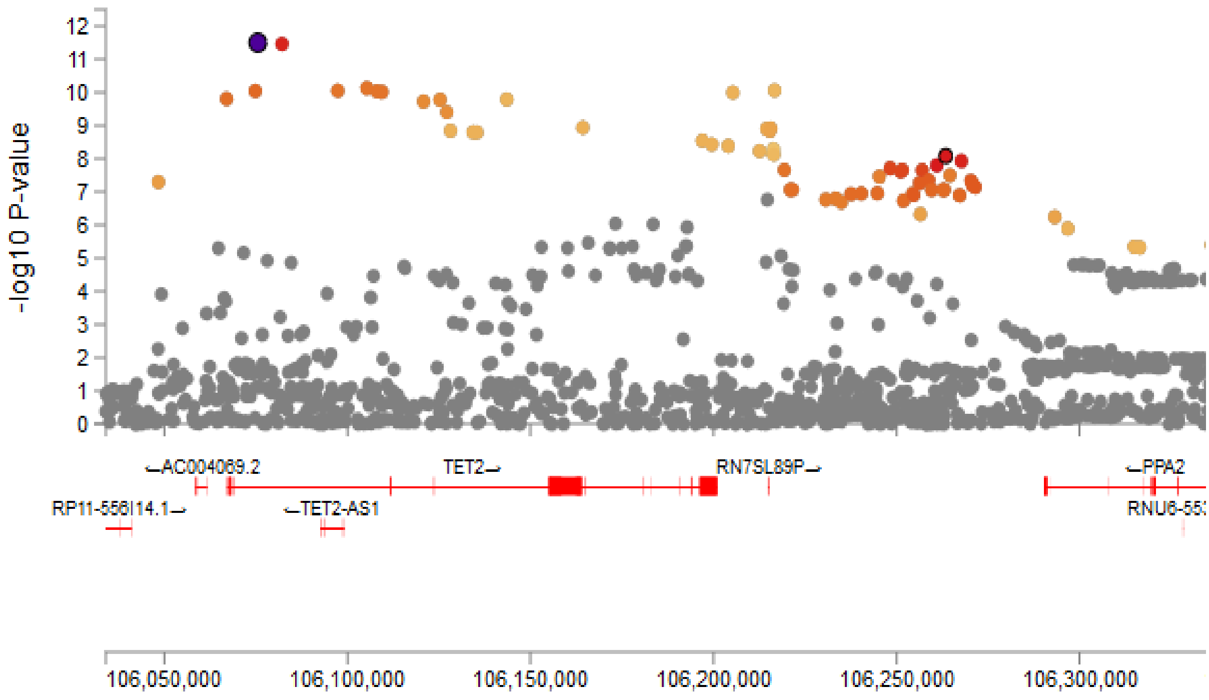

c. Locus 4B: Chr4:106048360-106335951

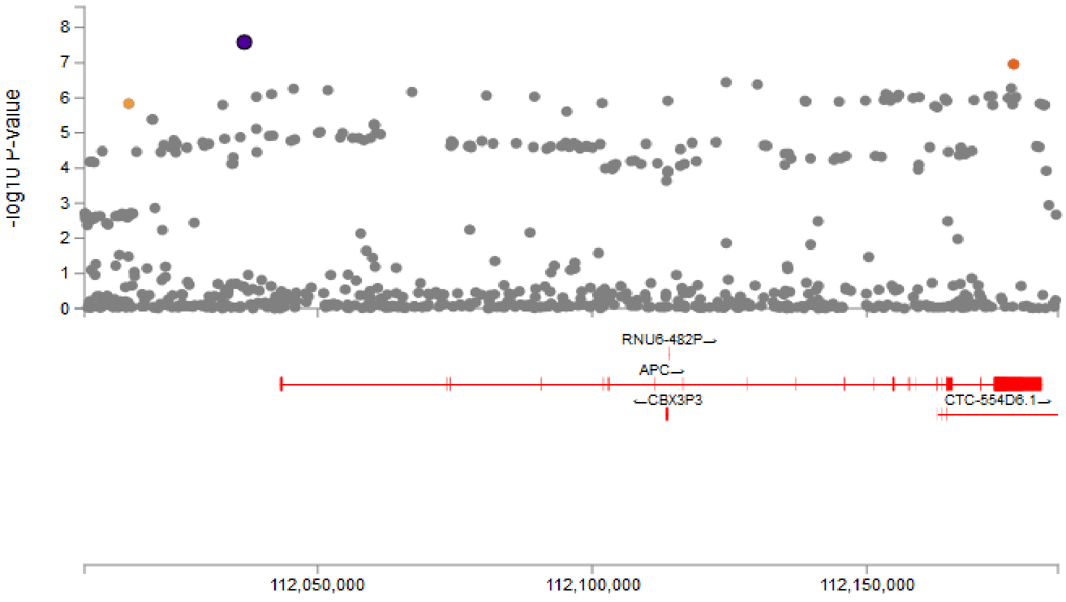

d. Locus 5A Chr5:112015555-112176756

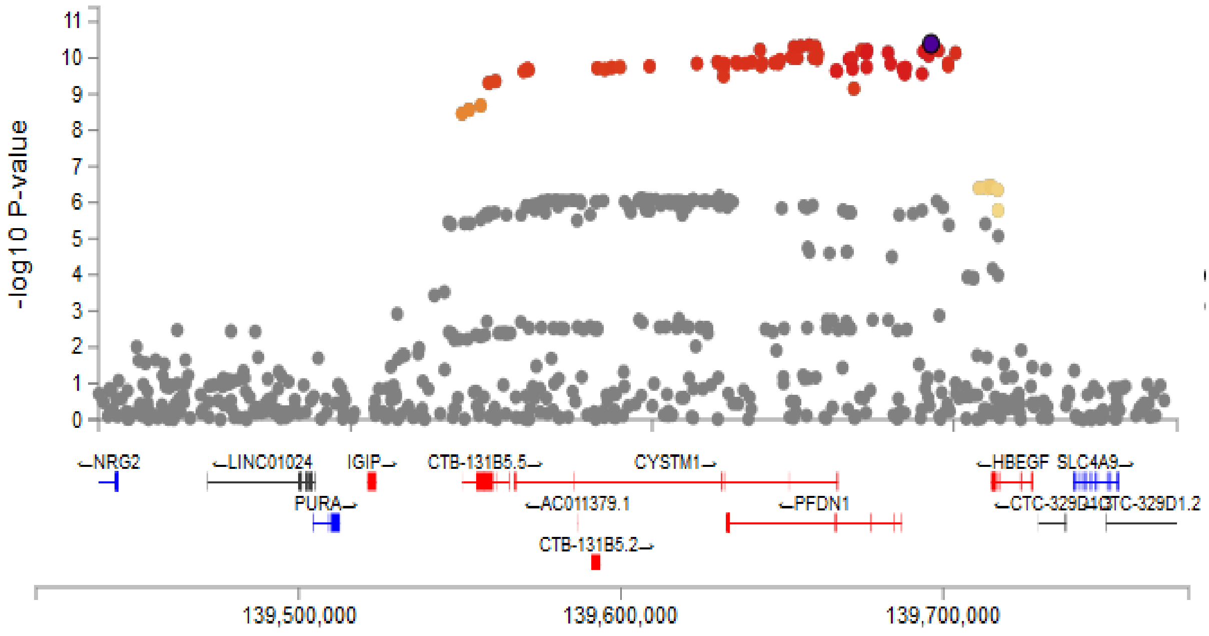

e. Locus 5B: Chr5:139517197-139714690

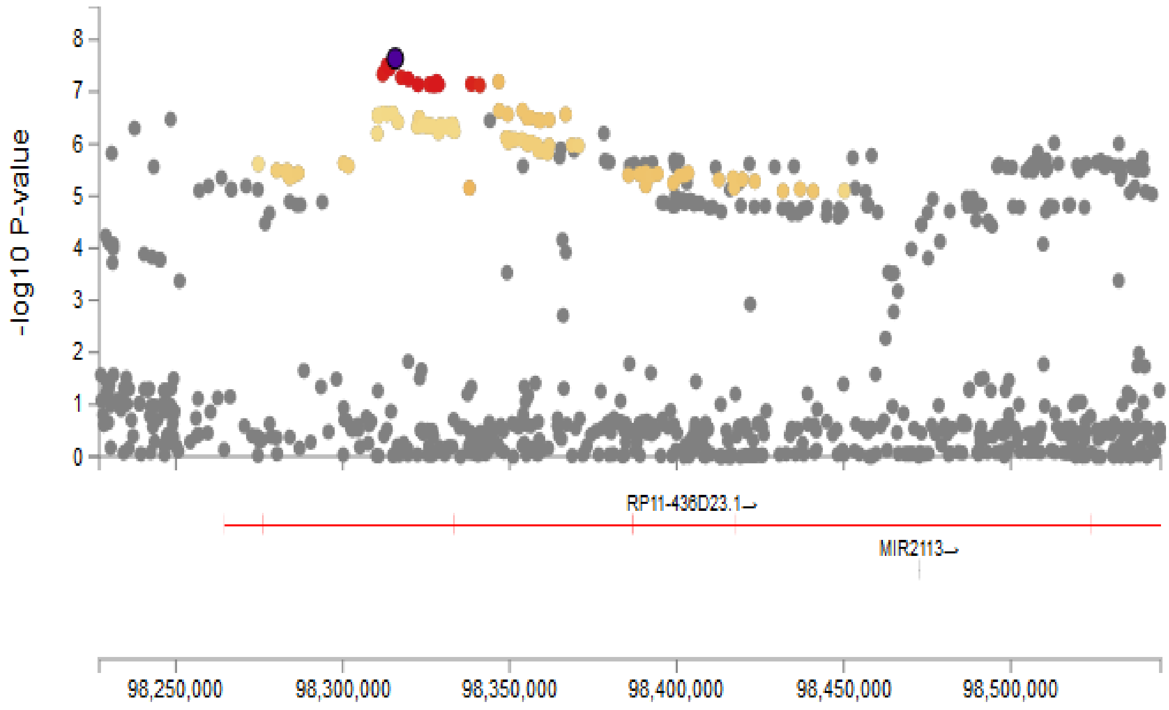

f. Locus 6: Chr6:98274701-98450190

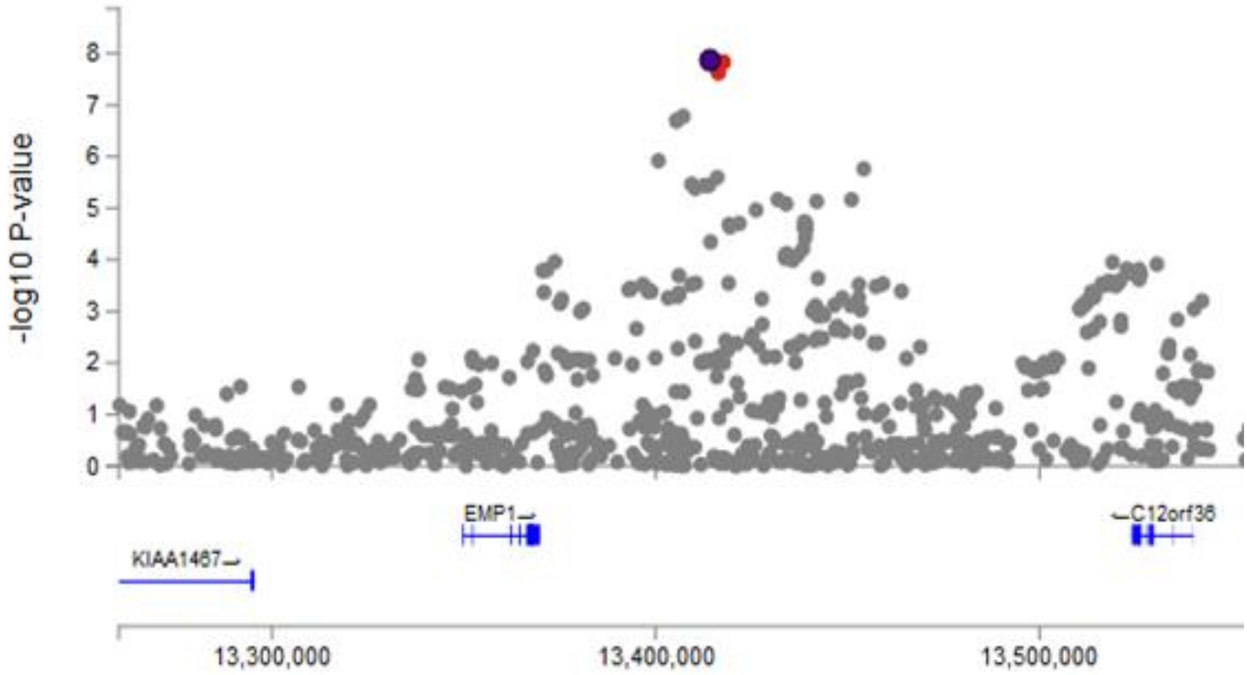

g. Locus 12A: Chr12:13414139-13417617

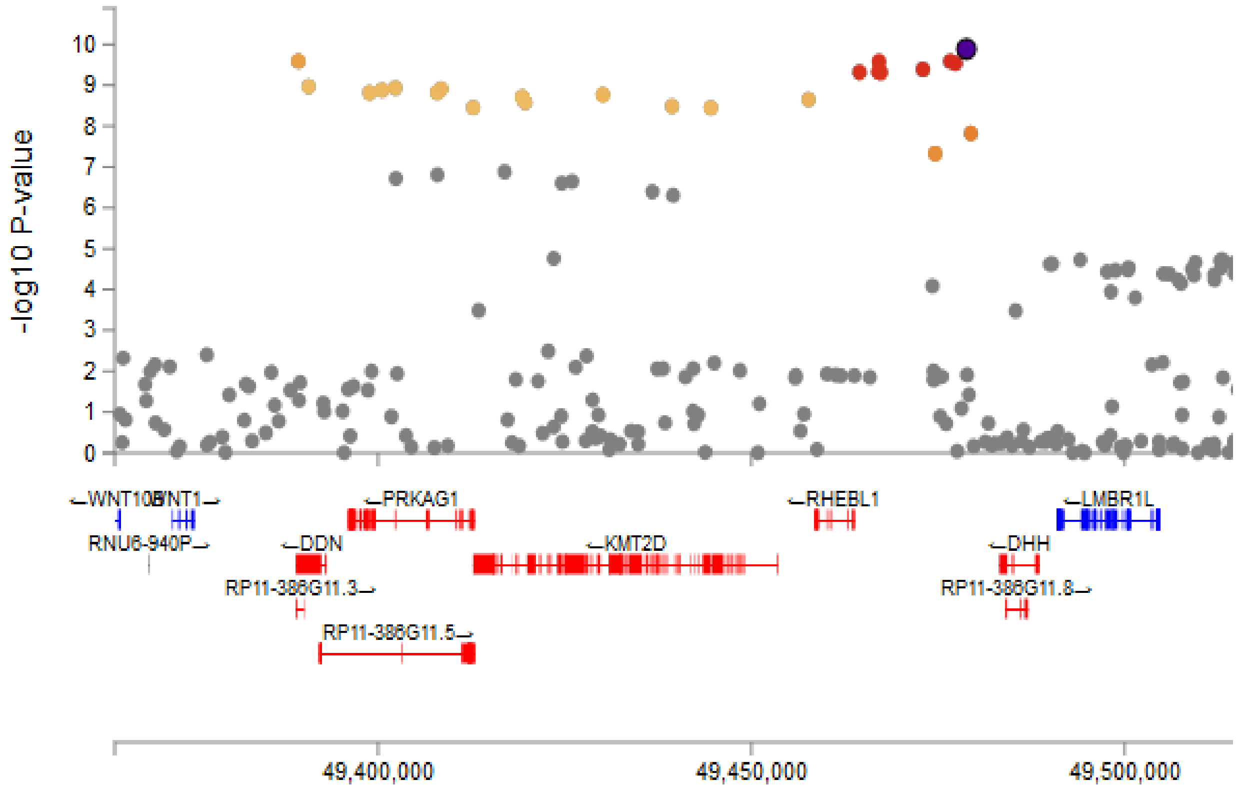

h. Locus 12B: Chr12:49387955-49479968

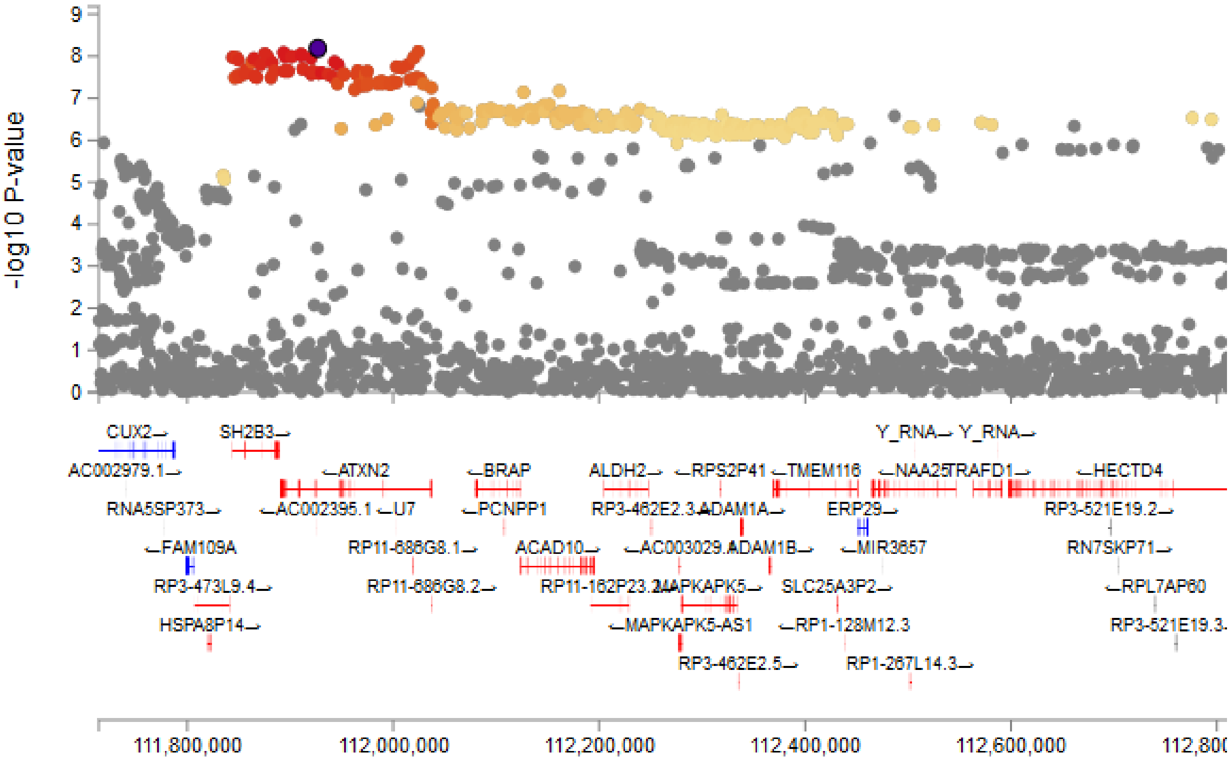

i. Locus 12C: Chr12:111818487-112817847

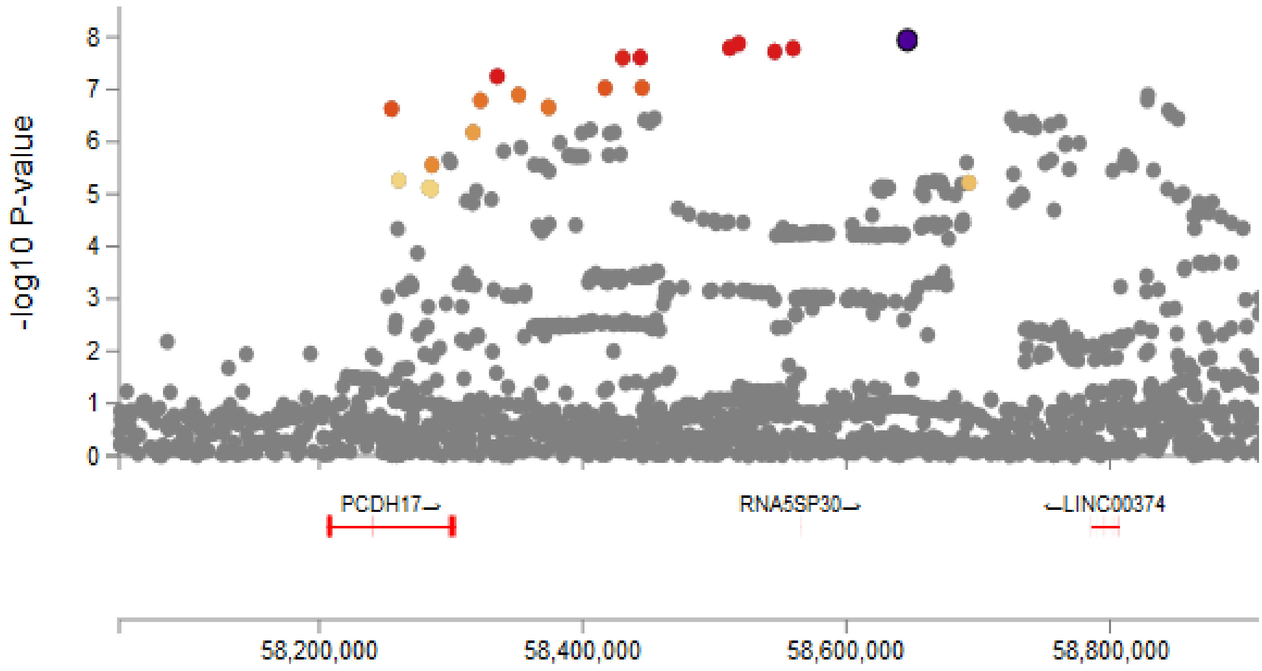

j. Locus 13: Chr13:58250322-58796832

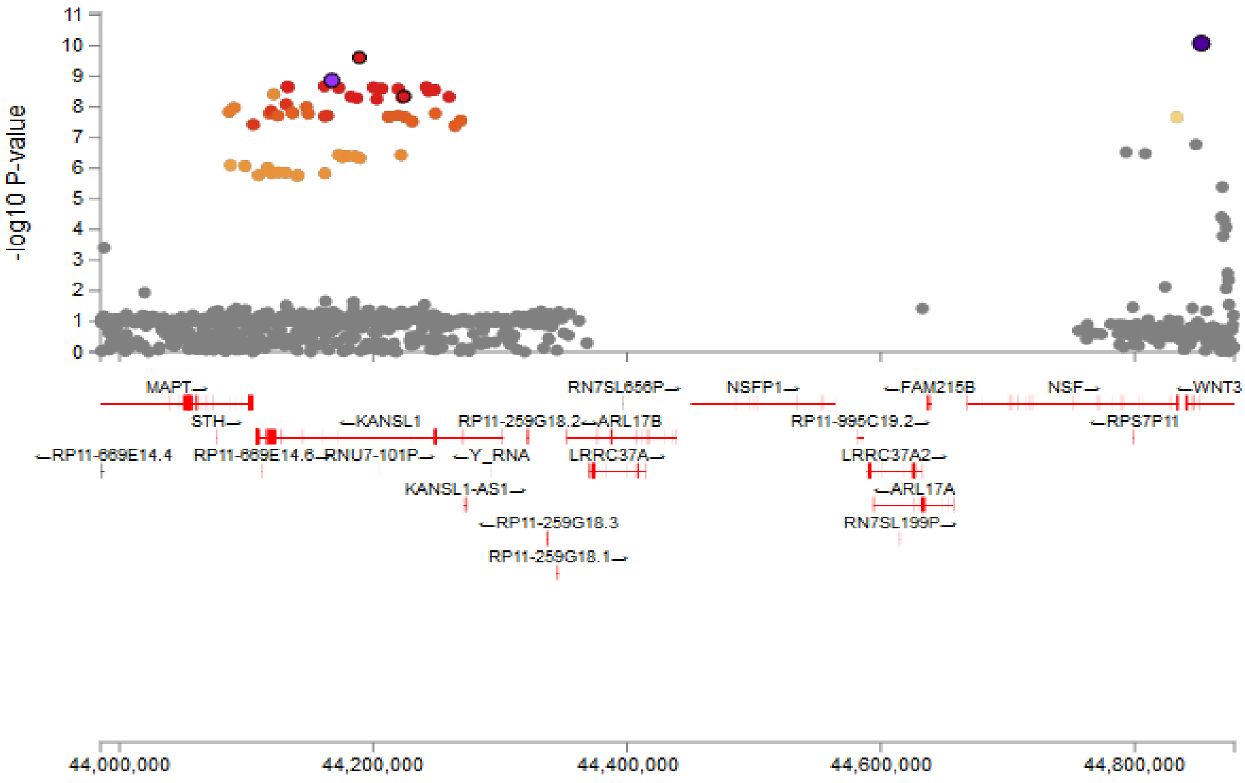

k. Locus 17: Chr17:44040184-44852612

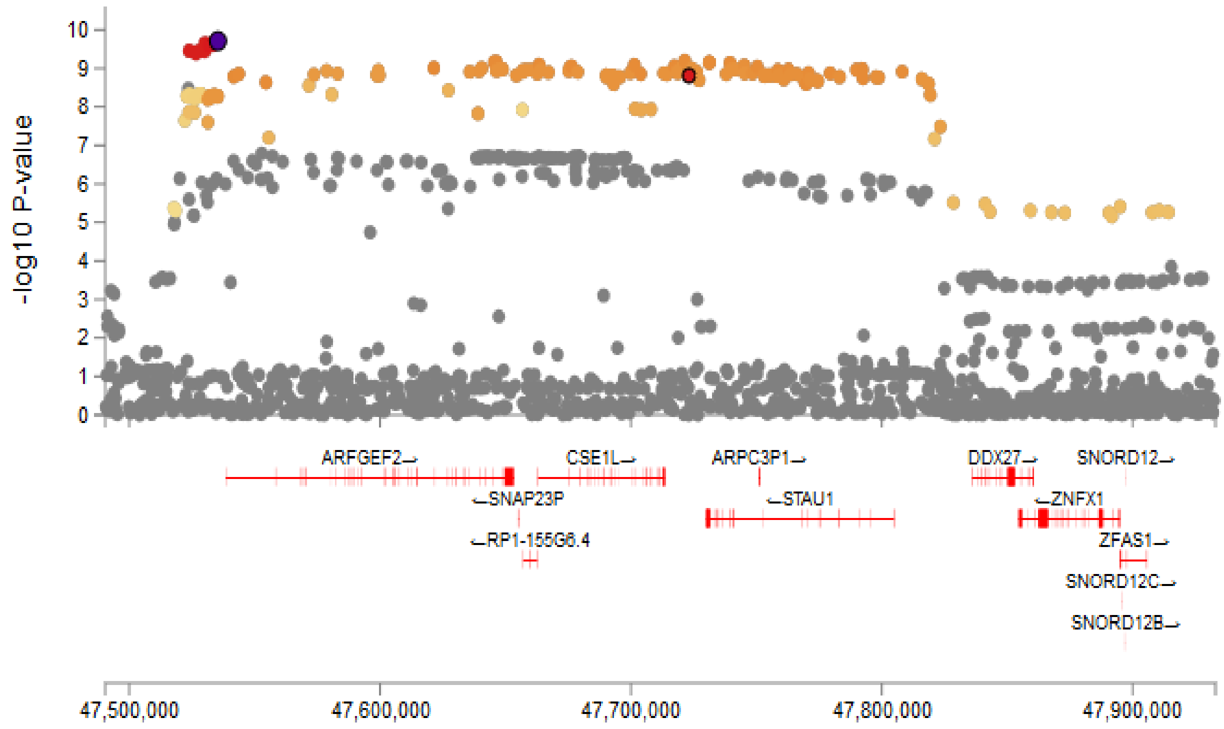

l. Locus 20: Chr20:47511792-47914180

Supplementary Figure S4: Circos plots of chromosomes that contain genome-wide significant loci. Genomic loci are highlighted in blue. Orange represents genes that are mapped by chromatin interaction and green represents eQTL mapping. If genes are mapped by both, they are highlighted in red. The dark blue portion of the inner circles represents the loci.

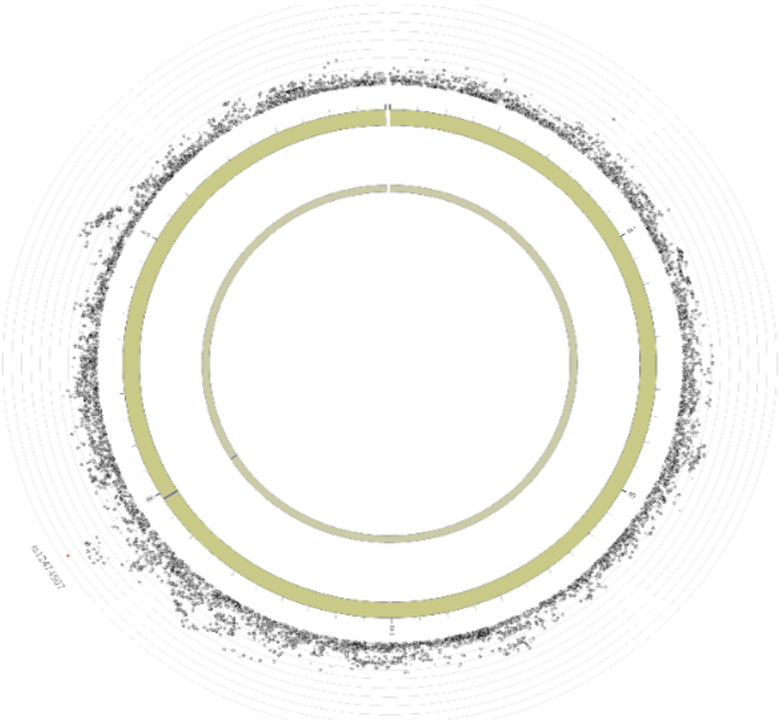

Chromosome 2

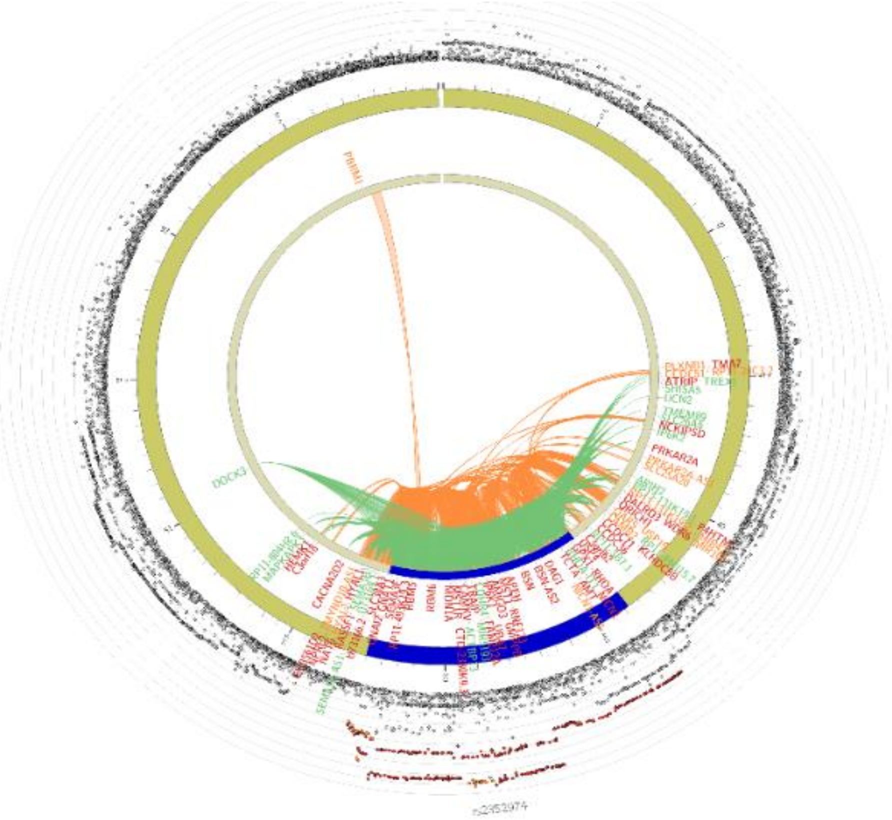

Chromosome 3

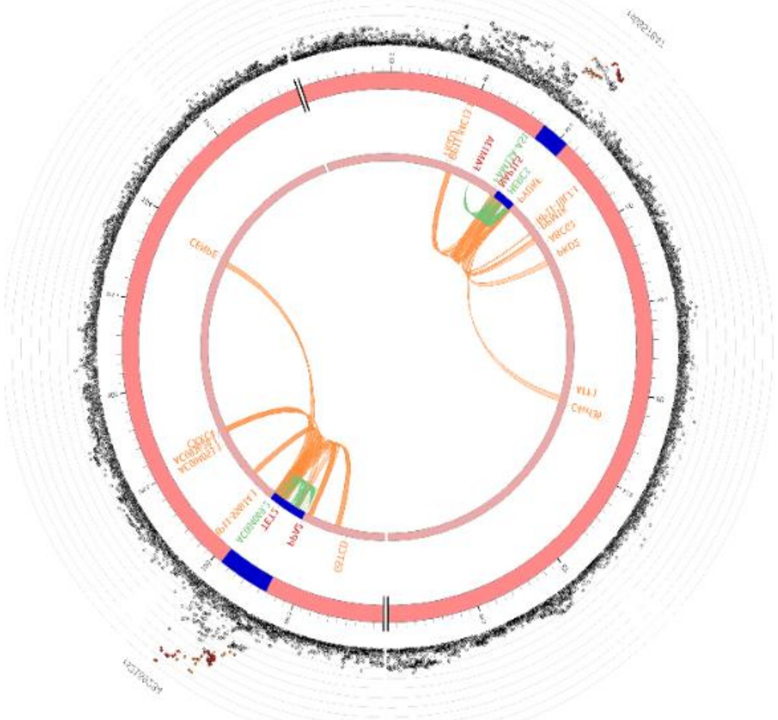

Chromosome 4

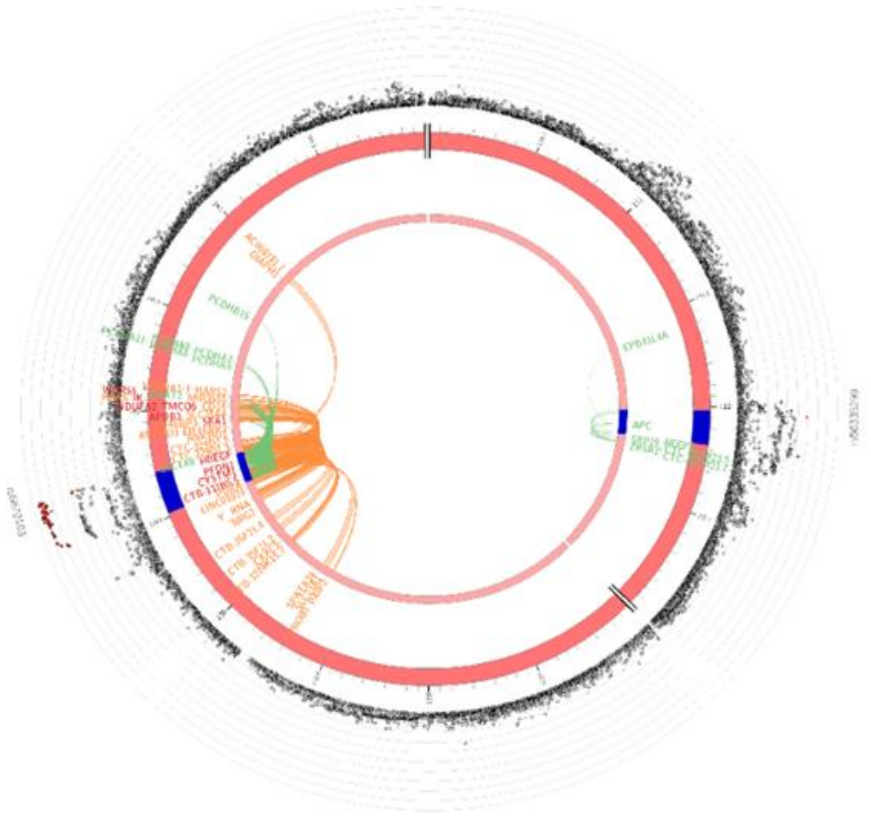

Chromosome 5

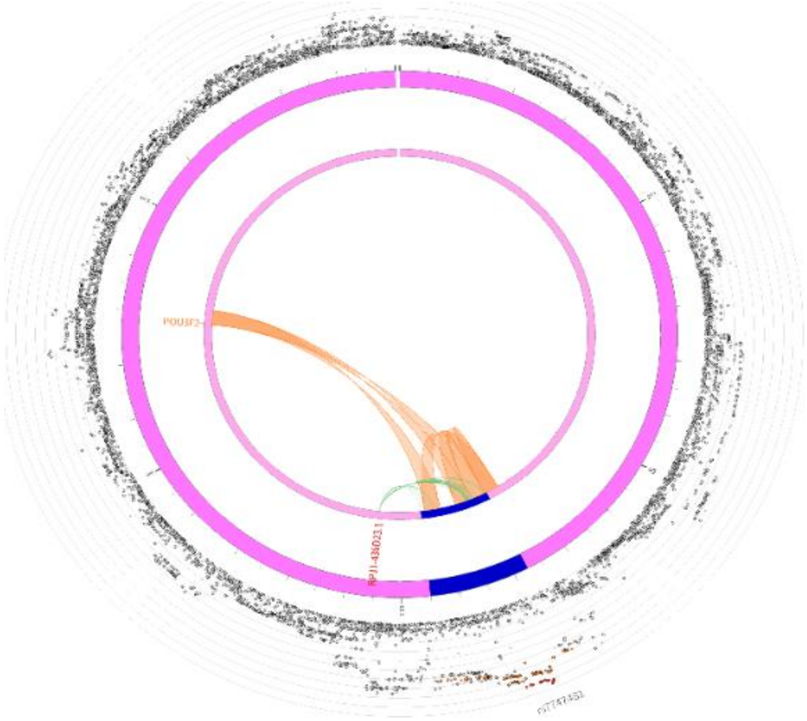

Chromosome 6

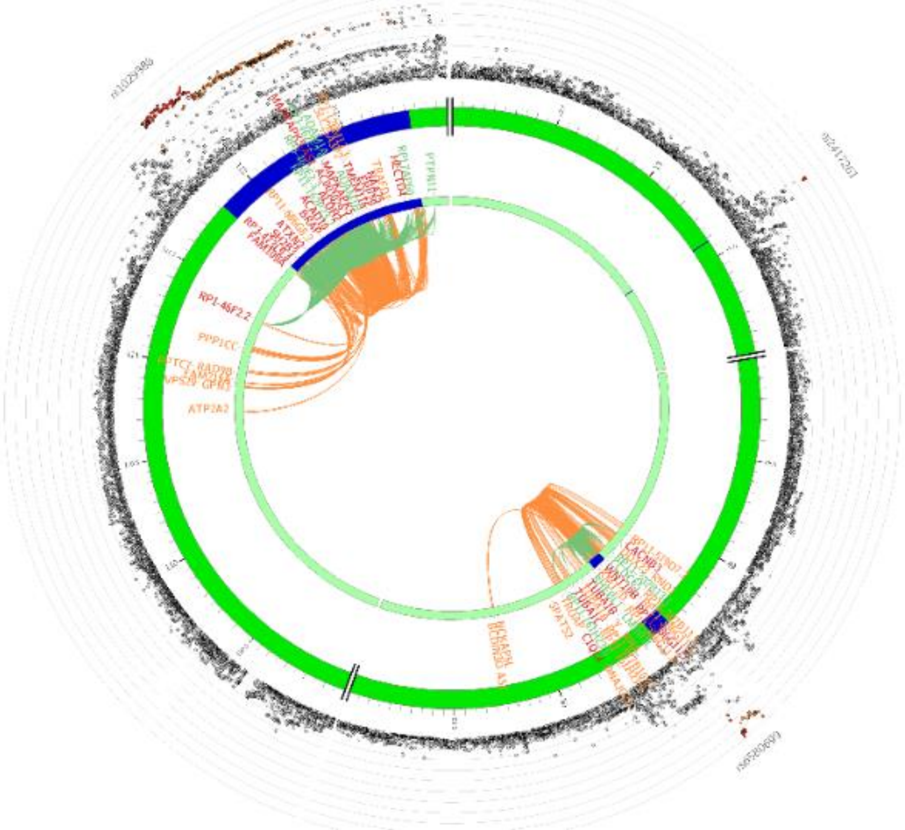

Chromosome 12

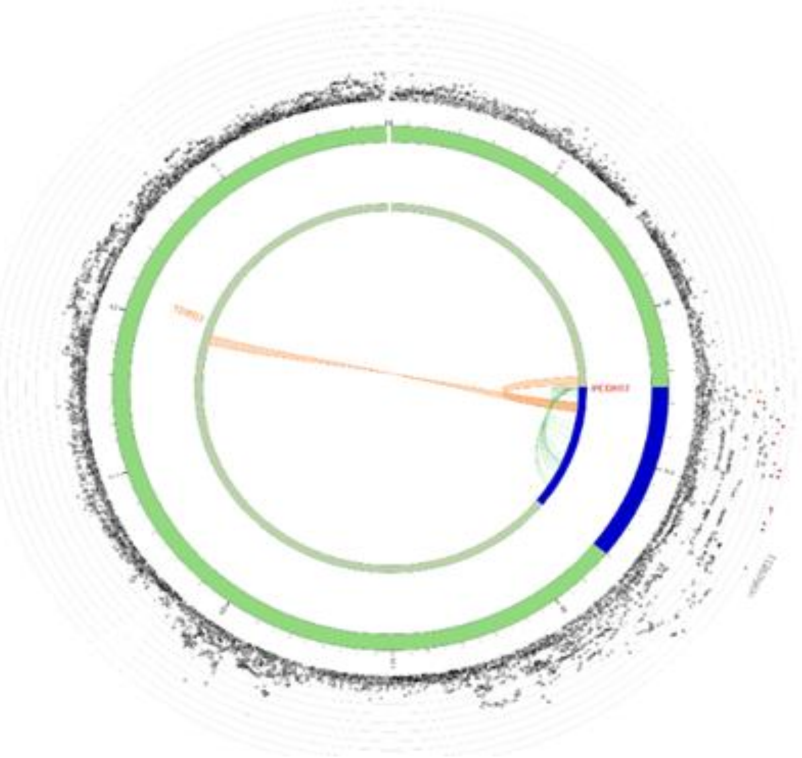

Chromosome 13

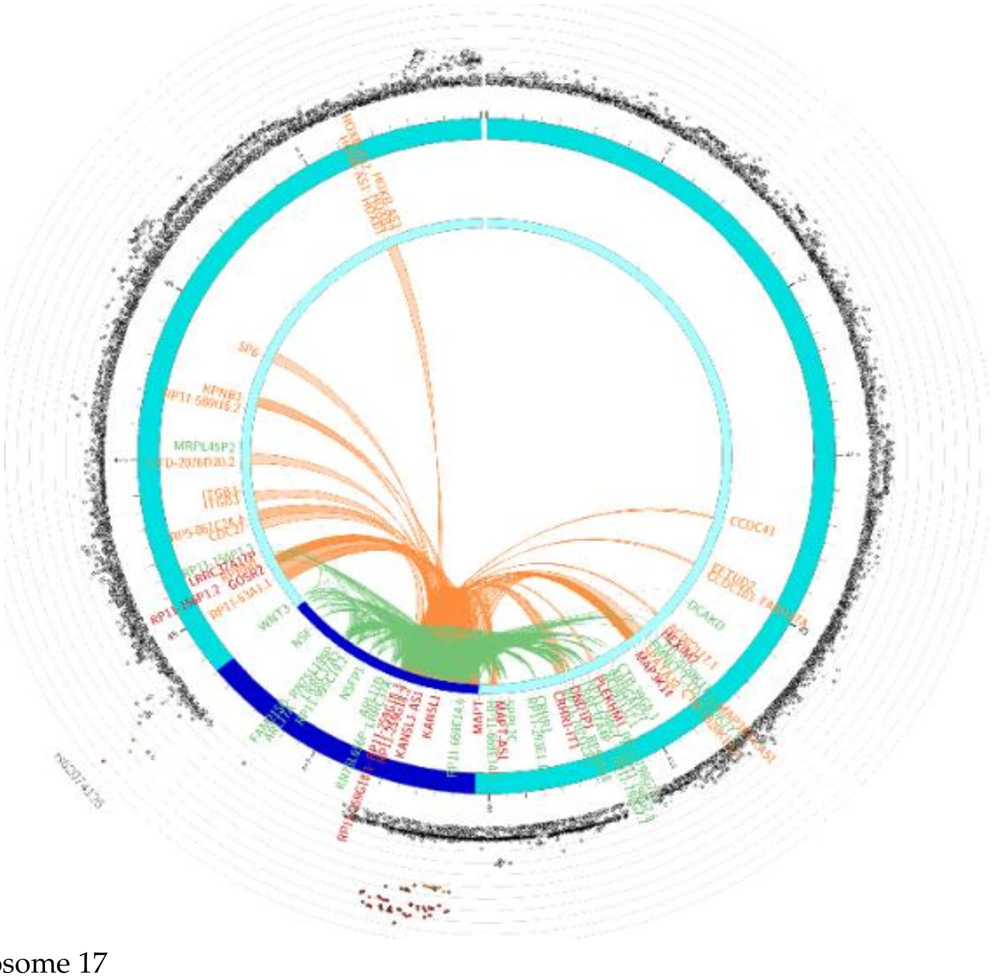

Chromosome 17

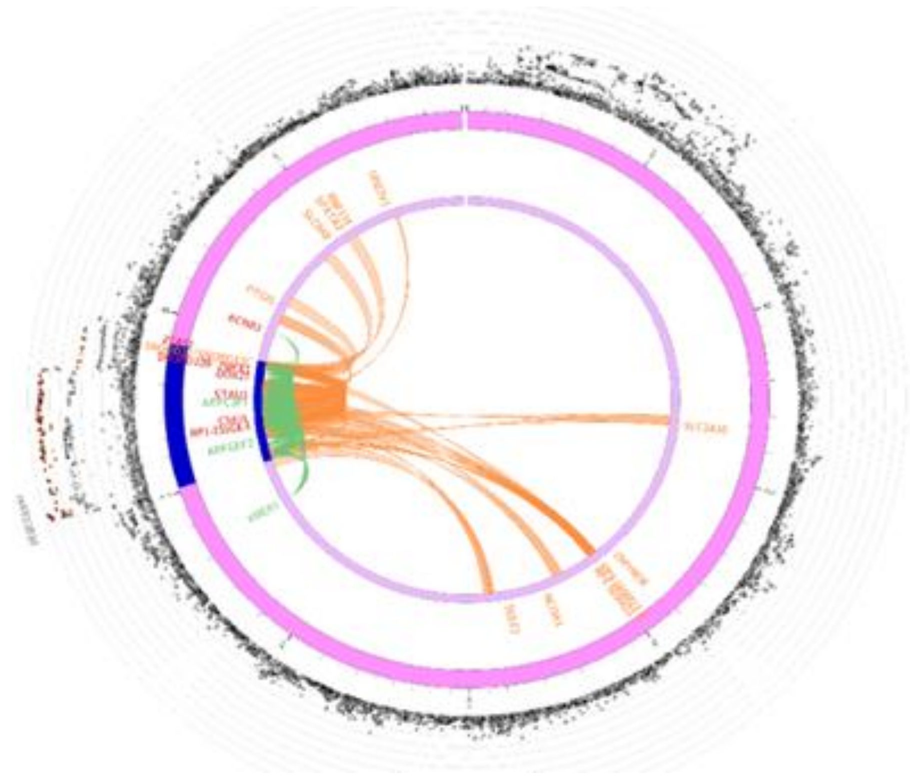

Chromosome 20

Supplementary Figure S5: Gene mapping of RT and comparison to *Resilience.* **a** Venn diagram of overlapping mapped genes by four strategies showing 27 genes were mapped by all four strategies for RT. **b** Venn diagram showing the overlap between the 27 prioritized genes from functional analysis of RT and the 33 prioritized genes from functional analysis of *Resilience.*

a

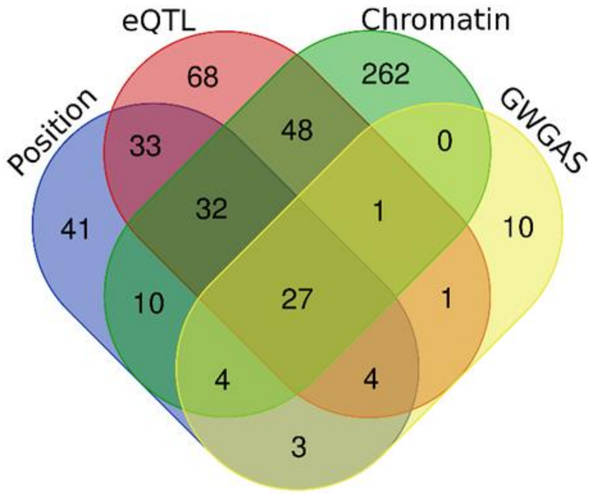

b

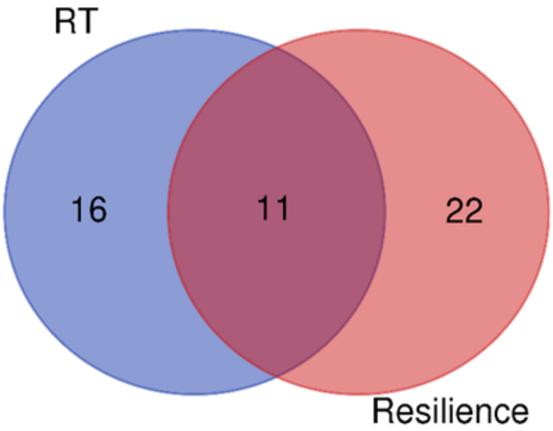

Supplement: Supplementary file 1 [file genes-13-00122-s001.zip › Supplementary_Figures.pdf]
